# Supplementary material for: A theoretical model of neural maturation in the developing chick spinal cord
Source: PLoS One. 2020 Dec 18;15(12):e0244219. doi: 10.1371/journal.pone.0244219 (PMC7748286; doi:10.1371/journal.pone.0244219)
Supplement: S1 File — (DOCX) [file pone.0244219.s001.docx]

## Supplementary methods

**Hill functions describing general regulatory interactions**

The Hill functions $H_{1}$,$H_{2}$,$H_{3}$, $H_{4}$ were used in the transcription and translation equations to describe one of four different types of interactions: (1) inductive, (2) repressive, (3) coordinated and (4) competitive. We generated transcription and translation equations specific for each factor by replacing $H_{1}$,$H_{2}$,$H_{3}$, and $H_{4}$ with the functions that represent the regulatory interactions reported experimentally.

INDUCTIVE INTERACTIONS. $H_{i}$, describes the input of one or multiple activators and can take three different forms.

$H_{i}$ for a single activator is of the form:

$$H_{i}=\frac{\left( \frac{A}{A_{c}} \right)^{a}}{1+\left( \frac{A}{A_{c}} \right)^{a}}$$

$H_{i}$ for two activators, when both activators are necessary to stimulate activity (Boolean AND), is of the form:

$$H_{i}=\frac{\left( \frac{A1}{{A1}_{c}} \right)^{a1}}{1+\left( \frac{A1}{{A1}_{c}} \right)^{a1}} x\frac{\left( \frac{A2}{{A2}_{c}} \right)^{a2}}{1+\left( \frac{A2}{{A2}_{c}} \right)^{a2}}$$

$H_{i}$ for two activators, when one activator is sufficient to stimulate activity (Boolean OR), is of the form:

$$H_{i}=\frac{\left( \frac{A1}{{A1}_{c}} \right)^{a1}+\left( \frac{A2}{{A2}_{c}} \right)^{a2}}{1+\left( \frac{A1}{{A1}_{c}} \right)^{a1}+\left( \frac{A2}{{A2}_{c}} \right)^{a2}}$$

, where, for all these equations, $A, A1, A2$ are the concentrations of activator proteins; $A_{c}, {A1}_{c}, {A2}_{c}$ are the respective Hill constants; and *a, a1, a2* are the Hill coefficient of cooperativity.

REPRESSIVE INTERACTIONS. $H_{i}$, describes the input of one or multiple repressors and can take three different forms.

$H_{i}$ for a single repressor is of the form:

$$H_{i}=\frac{1}{1+\left( \frac{R}{R_{c}} \right)^{r}}$$

$H_{i}$ for two repressors, when both repressors are necessary for downregulation (Boolean AND), is of the form:

$$H_{i}=\frac{1}{1+\left( \frac{R1}{{R1}_{c}} \right)^{r1}}x\frac{1}{1+\left( \frac{R2}{{R2}_{c}} \right)^{r2}}$$

$H_{i}$ for two repressors, when one repressor is sufficient for downregulation (Boolean OR), is of the form:

$$H_{i}=\frac{1}{1+\left( \frac{R1}{{R1}_{c}} \right)^{r1}+\left( \frac{R2}{{R2}_{c}} \right)^{r2}}$$

, where, for these equations, $R, R1, R2$ are the concentration of repressor proteins; $R_{c}, {R1}_{c}, {R2}_{c}$ are the respective Hill constants; and *r, r1, r2* are the Hill coefficients of cooperativity.

COORDINATED INTERACTIONS. $H_{i}$, describes the input of multiple activators and repressors working simultaneously through separate regulatory sites.

$$H_{i}=\frac{\left( \frac{A}{A_{c}} \right)^{a}}{1+\left( \frac{A}{A_{c}} \right)^{a}} x\frac{1}{1+\left( \frac{R}{R_{c}} \right)^{r}}$$

, where, for these equations, $A, R$ are the concentration of activator and repressor proteins; $A_{c}, R_{c}$ are the respective Hill constants; and *a, r* are the Hill coefficients of cooperativity.

COMPETITIVE INTERACTIONS. $H_{i}$, describes the input of multiple activators and repressors working simultaneously through the same regulatory sites.

$$H_{i}=\frac{\left( \frac{A}{A_{c}} \right)^{a}}{1+\left( \frac{A}{A_{c}} \right)^{a}+\left( \frac{R}{R_{c}} \right)^{r}}$$

, where, for these equations, $A, R$ are the concentration of activator and repressor proteins; $A_{c}, R_{c}$ are the respective Hill constants; and *a, r* are the Hill coefficients of cooperativity.

Hill constants measure the interaction strength between the activators/repressors and their target gene, and are defined as the concentration of the regulator at which the rate of gene activation or repression is half the synthesis rate maximum [1]. The hypothetical range for Hill constants is from zero to infinity, with low Hill values signify strong and fast regulatory activities while high Hill values signifying weak and slow regulatory activities (**Fig S1**).

## In situ hybridization

Analysis of gene transcription by *in situ* hybridization was done using digoxigenin (DIG)-labeled antisense RNA probes synthesized and hybridized using standard protocol [2], as previously described [3]. Briefly, embryos were harvested at the appropriate stage and fixed with 4% paraformaldehyde diluted in 1x PBS at 4^°^ C overnight. After a series of washes, embryos were exposed overnight in hybridization solution to DIG-labeled antisense RNA probes against *Pax6*, *Nkx1.2*, *Bra*, *Sox2*, *Cdx4 or Ngn2*. mRNA expression was detected using an Alkaline Phosphatase coupled Anti-DIG antibody (Roche) and developing embryos with nitro-blue tetrazolium salt (NBT, Thermo Scientific) and 5-bromo-4-chloro-3-indolyl-phosphate (BCIP, Biosynth) at room temperature until dark purple precipitate deposited revealing the areas of gene transcription. Post-development, embryos were washed with 1x TBST and then fixed in 4% PFA. Processed embryos were photographed using an AxioCam MRc digital color camera mounted on a Zeiss V20 Stereo microscope and processed using Adobe Photoshop (CC2017, Adobe) for size and resolution adjustment and figure preparation.

## References (Supplemental Methods)

1. Santillan M. On the Use of the Hill Functions in Mathematical Models of Gene Regulatory Networks. Math. Modelling Nat. Phen. 2008;3: 85-97.

2. Wilkinson DG, Nieto MA. Detection of messenger RNA by in situ hybridization to tissue sections and whole mounts. Meth. Enzymol. 1993;225: 361-373.

3. Joshi P, Darr AJ, Skromne I. CDX4 regulates the progression of neural maturation in the spinal cord. Dev. Biol. 2019;449: 132-142.
