# Supplementary figures and images for: A theoretical model of neural maturation in the developing chick spinal cord
Source: PLoS One. 2020 Dec 18;15(12):e0244219. doi: 10.1371/journal.pone.0244219 (PMC7748286; doi:10.1371/journal.pone.0244219)

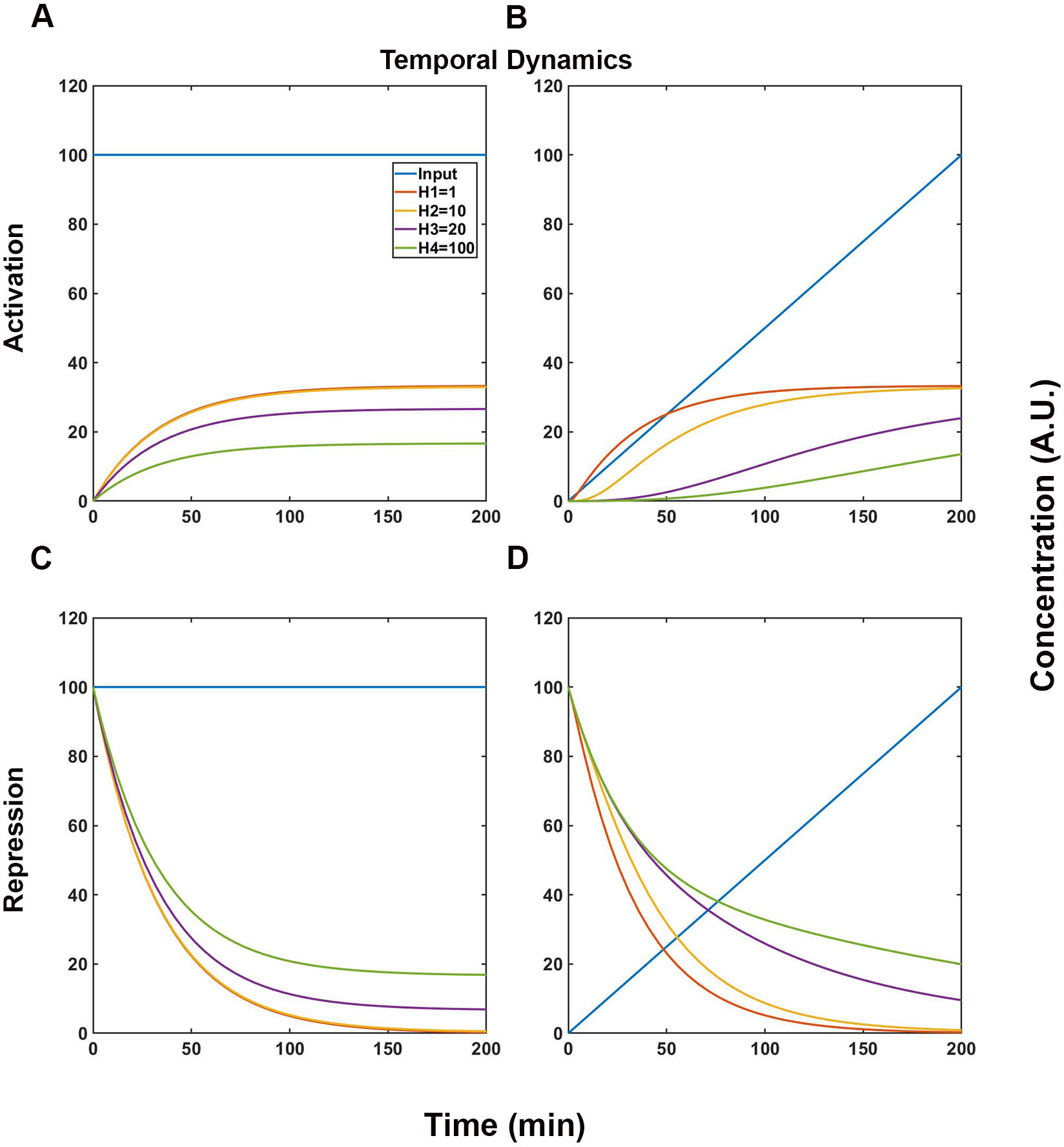

Supplement: S1 Fig — Temporal response of targets with different Hill constants to activators and repressors. Inputs are shown in blue and targets with different Hill constants are color coded: H1 = 1, orange; H2 = 10, yellow; H3 = 20, purple; and H4 = 100, green. (A-B) For activators, constant (A) and graded (B) inputs induce targets with smaller Hill constants to higher levels than targets with larger Hill constants. (C-D) For repressors, constant (C) and graded (D) inputs reduce targets with smaller Hill constants to lower levels than targets with larger Hill constants. With graded inputs (B, D), larger Hill constants also cause temporal delays in response. (TIF) [file pone.0244219.s002.tif]

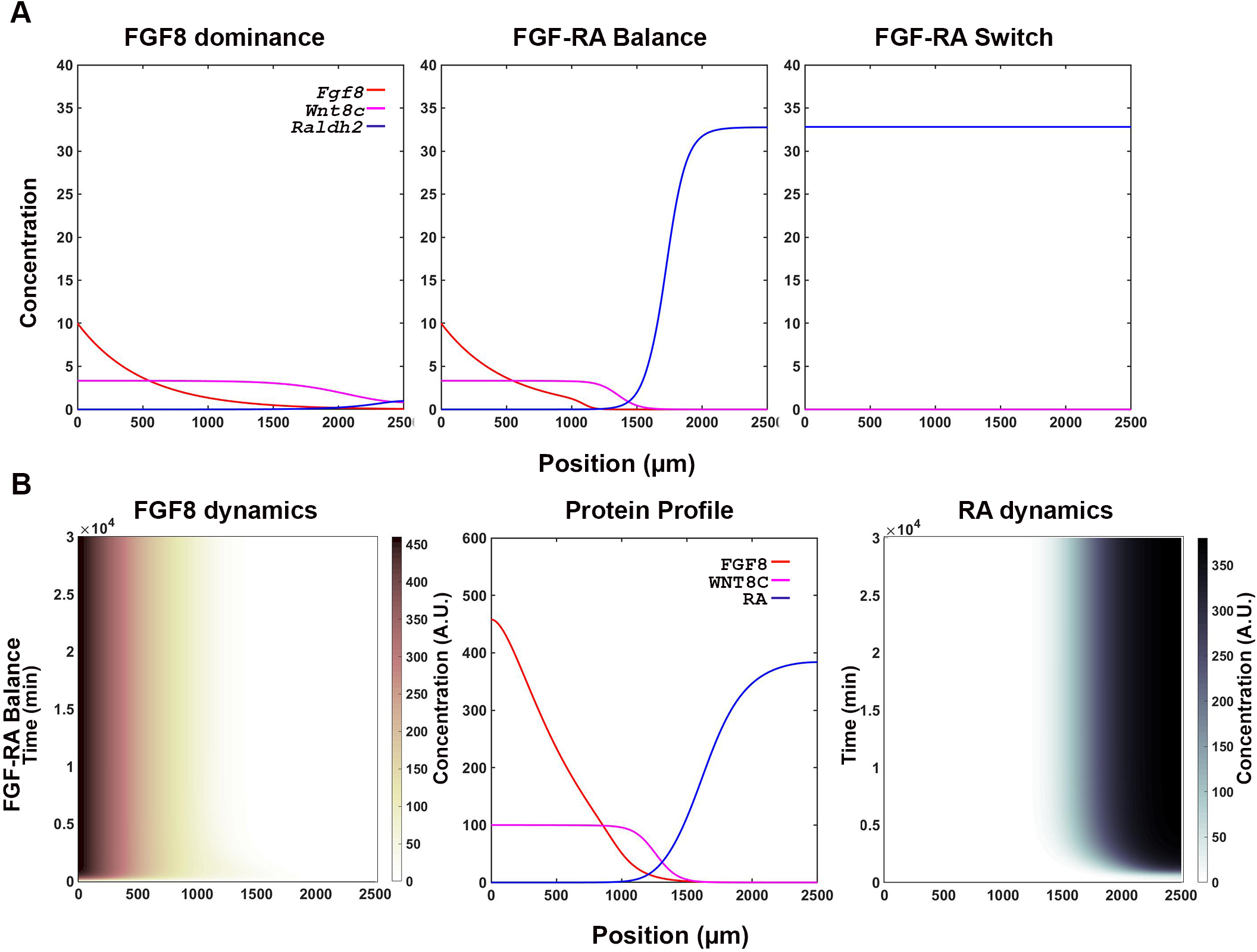

Supplement: S2 Fig — (A) Profiles of mRNA transcripts at t = 6000 min associated with production of signaling molecules. Transcript and protein profiles are similar (Fig 2C left panels). (B) Signaling molecule profiles are stable over longer simulation times. An FGF-RA balance simulation that was run for t = 30,000 min produced the same profile than a simulation that was run for t = 6000 min (Fig 2C middle row). (TIF) [file pone.0244219.s003.tif]

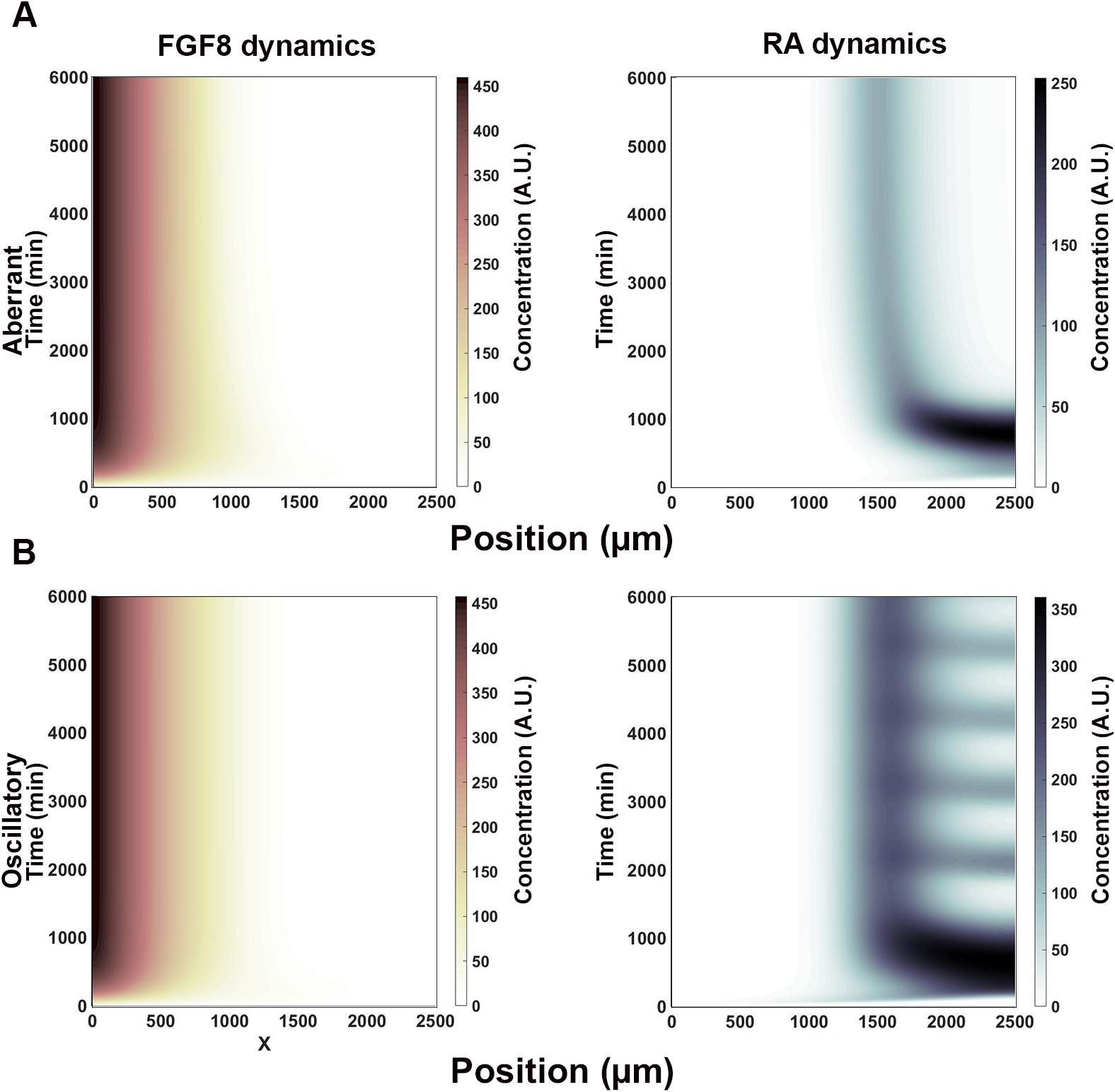

Supplement: S3 Fig — Reducing RA’s positive effect on Raldh2 transcription (H = 300 instead of 50) results in aberrant RA, but not FGF, distribution. (A) Under these conditions, when FGF affinity to repress Raldh2 is strong (H = 2 instead of 10), a peak of RA production forms at a position in the field where the FGF-RA switch would have occurred (1500–2000 μm). (B) When RA repression of Fgf8 transcription is weakened (H = 20 instead of 1), RA production oscillates in the region of cell differentiation (>1500 μm). (TIF) [file pone.0244219.s004.tif]

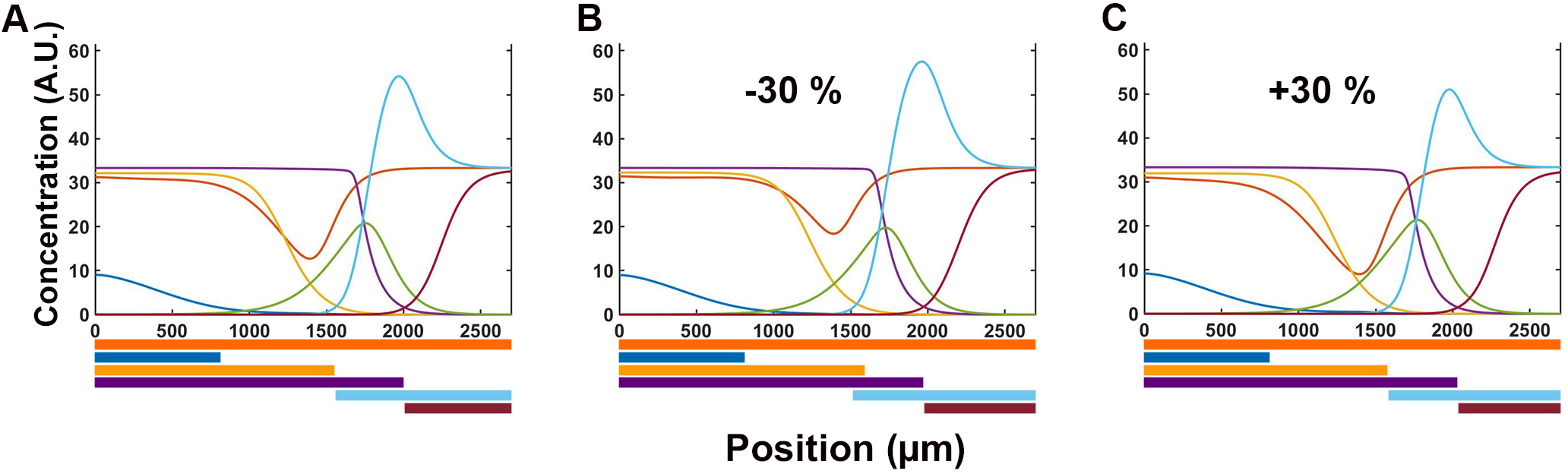

Supplement: S4 Fig — Changes in the strength of interactions between transcriptional factors does not drastically affect the transcriptional domain profile. (A) Original transcription profile as shown in Fig 4F. (B, C) Reducing (B) or increasing (C) all the Hill constants in the interaction network by 30% does not significantly change the spatial profile of gene transcription. (TIF) [file pone.0244219.s005.tif]
