# Supplementary material for: A theoretical model of neural maturation in the developing chick spinal cord
Source: PLoS One. 2020 Dec 18;15(12):e0244219. doi: 10.1371/journal.pone.0244219 (PMC7748286; doi:10.1371/journal.pone.0244219)
Supplement: Attachment — Submitted filename: Response to Reviewers.pdf [file pone.0244219.s008.pdf]

UNIVERSITY OF RICHMOND

Isaac Skromne, Ph.D.
Department of Biology
University of Richmond
138 UR Drive – B322
Richmond, VA 23173
Phone: 804-289-8235
iskromne@richmond.edu

October 19, 2020

Dear PLOS ONE Editor and Reviewers,

Please find enclosed the *revised* manuscript PONE-D-20-22620 entitled "A THEORETICAL MODEL OF NEURAL MATURATION IN THE DEVELOPING *CHICK* SPINAL CORD", where we analyze the individual and combined functions of signaling and a transcription factor networks in the maturation of spinal cord neural precursors in the trunk region of the chick embryo. We want to thank the reviewers for their time in reading the manuscript and providing positive feedback. We have incorporated most of their comments into the manuscript, as we believe they improve the clarity of the text and better contextualize our findings. Most of the concerns raised by the reviewers centered in justifying the incorporation or exclusion of molecular signals in our model, and the discussion of "gradual patterning and canalization" theories. We have directly addressed these and other tissues raised by the reviewers and fully explain them in the following pages. It is worth highlighting that the reviewers did not provide critique on the mathematical analysis and the results obtained, as well as their interpretation. It is our hope that our revisions satisfy the reviewer's concerns. We believe that the work conforms to *PLOS ONE's* high quality standards and that the findings reported in this work will be of interest to both mathematicians and developmental biologists.

Sincerely

Isaac Skromne
Assistant Professor of Biology

RESPONSE TO REVIEWERS

General issues raised by both reviewers

1. Reviewer 1 and 2 had a shared concern regarding the our use of ordinary and partial differential equations. Reviewer 1 states in his/her/their question 2 that "for someone who is unfamiliar with modelling biological processes with differential equations, it may be difficult to initially understand why a combination of partial differential equations are necessary to input into an ordinary differential equations. A brief background regarding the subsequent role of partial and ordinary equations can help disseminate your findings to the lay population of biologists." Reviewer 2 states in his/her/their question 1 that "the authors highlight the models used in great detail, which may be overwhelming to some readers."

(RESPONSE) We have added language in the manuscript explaining the use of ordinary and partial differential equations, and clarified their use in the signal and transcription factor network (page 7, first paragraph). Both equations follow the same form, except that partial differential equations take into consideration diffusion when calculating the local concentration of a secreted factor in space. So, ordinary differential equations were used to calculate local concentrations of non-diffusible molecules (e.g., mRNA and intracellular proteins), and partial differential equations for secreted molecules (e.g., signaling factors). In the signaling network, both types of equations were used to calculate transcription levels (ordinary equations for mRNA) and signaling factor levels (differential equations for diffusible molecule). In the transcription factor network only used ordinary differential equations.

Specific issues raised by Reviewers 1

1. This paper is mainly based on the theory of derivation of spinal neural progenitors from "Neuromesodermal progenitors" (NMP) that is first introduced by Mina Gouti and is now continued by her lab. Although there is robust experimental evidence for this model, another model for derivation of spinal neural progenitor cells

is that of gradual patterning and canalization. The authors need to explain these two models and discuss how their mathematical model fits to any or both of those experimental models.

(RESPONSE) We thank the reviewer for bringing to our attention the gradual patterning and canalization model of neural progenitor cell maturation. While Reviewer 1 is correct in identifying Mina Gouti's work as the original impetus behind testing CDX function in neural progenitor cell maturation, our model does not rule canalization as a mechanism for gradual pattering. In fact, as explained in the revised text, part of our analysis support a role for the networks in canalization. Canalization is an emergent property of systems that allow cells to remain in their developmental trajectories in the face of genetic or environmental perturbations [1]. The robustness of the combined signaling and transcription factor network to withstand noise and other perturbations is an emergent property of the system that was not apparent when analyzing each network individually, and therefore, a potential good example of canalization. In the revised text, we now discuss canalization in the introduction, results and discuss sections of the manuscript (lines 53, 565 and 666).

2. Question addressed above in the section "general issues raised by both reviewers."

3. The modeling is heavily based on the expression level of "Cdx4" that the authors found contributed in the their last experimental paper (Joshi et al. 2019). It seems that in the current paper the authors want to justify the importance of Cdx4, but ignore other important transcription factors like Hox genes, and Cdx1.

(RESPONSE) Our previous work and the current manuscript focuses on the activity of CDX4 in the caudal neural plate of the chick embryo because this is the *only* CDX family member transcribed in chick between 10-18 somite stage of development [2]. This was a conscious decision, as we wanted to have a clear understanding of the system without having to deal with the redundant activities of CDX1 and CDX2

obscuring the results. We have now included this rationale in a new sub-section of the Methods section entitled Model Outline (see line 139).

HOX transcription factors were not included in our model because their function in the spinal cord is to provide axial identity of neural precursors (*Hox* groups 5-12) or to terminate axial elongation (*Hox* group 13) and, as far as we know, there is no evidence supporting a role of these genes in neural precursor cell maturation. Work from the Capecchi and Deschamps lab have shown that triple mutant mouse embryos for Hox paralogous group 8, 9, 10, or 11 have a full but transformed number of vertebrae in the spinal column, and what appears to be a normal spinal cord. Thus, it is not the production or maturation of neural tissue what is affected in these triple mutants, but the axial identity of the derived tissue. *Hox* 13 genes are indeed involved in axial termination, as shown by the groups of Deschamps and Mallo. This activity, however, takes place during the tail bud stages of development, which is *after* the developmental stages that our model is emulating (chick tailbud stages start between 16-20 somite stage of development; [3]). Nonetheless, we have now included a short discussion of HOX group 13 gene function in our revised manuscript (see lines 620 and 700).

4. The assumptions used for the differentiation of spinal neural progenitor cells in this paper are over-simplified. The purpose of mathematical modeling is to predict complex biological/developmental events. Oversimplifying the assumptions and ignoring some details and pathways in favor of other factors is contradictory to the purpose of mathematical modeling.

(RESPONSE) The model attempts to mirror as close as possible experimental observations, without oversimplifying or assuming interactions that experimental data hints to but have not been demonstrated rigorously. All the interactions (edges) of the network are solidly supported by experimental evidence, either ours or others. We decided to err on the side of caution and not add interactions where the experimental data

is ambiguous or parametric values are not available in chick. As Reviewer 1 indicates in questions 7 and 8 when asked about the species used (chick, see below for full reply), we are trying to model a natural phenomenon, not a mythological Chimera. With respect to the mathematical representation of the interactions and the parametric values used as inputs, we are following accepted standard in the field (see, for example, [4]). In the Methods section we make reference to other mathematical clock-and-wavefront models of paraxial mesoderm segmentation or neuronal *differentiation* in chick as a guide to our model [4, 5], as ours is the first attempt to model neural progenitor cell *maturation* in the spinal cord. The assumptions made in our model are similar to assumptions made in all those other models.

5. The manuscript does not consider the BMP morphogen gradients of growth differentiation factor 11 (GDF11) in the AP-axis nor BMP DV-axis developmental modelling. Also, the effect of Notch signaling in patterning is heavily ignored. TGF-b gradient, DKK1, FRZB, GDF7 and … factors should be considered in the equation. The assumptions used for the differentiation of spinal neural progenitor cells in this paper are over-simplified.

(RESPONSE) Modeling the interactions of every single molecule known to pattern the nascent spinal cord would be an important and incredible feat that, at this moment, it is out of reach. Not only we *don't* know *all* the regulatory interactions between signaling factors directing the specification, maturation and differentiation in all the embryonic axes (rostral-caudal (FGF, WNT, RA, GDF11, etc.), dorsal-ventral (SHH, WNT, BMP, GDF6, etc.), apical-basal (NOTCH, etc.)), but we also don't know the parametric values associated with these interactions. Similar argument can be made for all the components of the gene regulatory networks (e.g., LIM, HOX, NKX, PBX, CDX, NGN, etc.). One important consideration when modeling these interactions is that, for each additional molecule included in the model, the number of the

equations that need to be solved simultaneously increases exponentially. At this moment, this feat is outside the scope of our work.

Two important signaling molecules that were not discussed in our model but are now including in our text are GDF11 and NOTCH. We are now providing explanations as to why GDF11 and NOTCH were *not* included in our model. There reason for not including GDF11 is that this factor functions in terminating axial elongation in mouse once the tailbud has formed [6, 7], an event that in chick takes place around the 16-20 somite stage of development [3], right *after* the events being modeled in our work. With respect to the NOTCH pathway, this pathway was not included in our model despite evidence of a role in neuromesodermal cell proliferation [8] because parametric values are not available for these interactions in chick. Our numerous experimental attempts to determine the genetic interactions between NOTCH and CDX4 have been inconclusive (data available upon request). The rational for not including GDF11 and NOTCH in our model is explained in the Model Outline and Discussion sections (see lines 140, 618 and 657).

6. To simplify their model, the authors consider the "most influential" factors FGF8/WNT8C to control FGF/WNT signaling in development. Depending on the species, different factors, such as FGF17, FGF18, FGF4 or WNT3A contribute to the morphogen regulation. These factors should also be considered when making a reliable predictable modeling.

(RESPONSE) We regret the use of the term "most influential" factor and we have eliminated the term in the manuscript. The model is based on the factors that in our model organism, the chick (see answer to next questions), have been shown experimentally to regulate neural progenitor maturation processes (FGF8, WNT8C and Retinoic Acid). We are NOT cherry picking the data; we are using the data that is available.

7. The authors do not specify the species that they are matching their signaling gradients to in their equations.
8. The authors should mention, more clearly, which species you are trying to model developmentally. The majority of their references seem to refer to either the chick or mouse, but different experimental evidence exists for either species, which may contradict, or have missing information, based on species variation.

(RESPONSE to 7, 8) We apologize to Reviewer 1 for the confusion that the lack of clarity in our text has generated. We have taken several measures to clarify the species used and the origin and justification of the parametric values used in the model.

- The model is based on data obtained in the chick embryo between 10-18 somite stage of development. At these stages, the embryonic axis is elongating at a relatively constant velocity, and the caudal neural plate has a relatively constant size [9]. We have explicitly stated the species used in the title of the work and throughout the manuscript.
- Most all parametric values used in the model are based on information obtained from chick embryos between 6-18 somite stage of development. References to these values are indicated in the text at the appropriate places. When an essential parametric value is not available in the chick, we have explicitly indicated the source of the value (e.g., cell culture systems, mouse embryos).
- We have added a new paragraph in the Future Perspective section of the manuscript addressing how our model in chick could inform neural maturation processes in other species (see line 679).

9. Simplification of morphogen gradients are only regulated by other protein-protein interactions dynamics. The paper fails to consider in their mathematical modelling how different neural cell fate transcription factors may affect these signaling ques during development.

(RESPONSE) Reviewer 1 brings up an important point that, unfortunately, cannot be addressed at the moment due to the scarcity of data regarding how neural transcription factors affect signaling ques. We recognize

that most information regarding gene regulatory networks focus in understanding how signaling factors regulate the expression of transcription factors specifying cellular states, and not the other way around. Some of this information is available in other systems, but not in the caudal neural plate. For example, published work has identified some of the transcription factors regulating FGF and Wnt expression in the midbrain/hindbrain (MB/HB) boundary or apical ectodermal ridge of limbs. We felt, however, that is not appropriate to use MB/HB or limb information in our model, as the transcriptional regulation of signaling factors in all these tissues may be different. One additional possibility that needs to be considered is that the transcription factors may work as a competence factor for the transduction of the signal. That is, in the absence and in the presence of a transcription factor, a cell responds to the same signal in different ways. Given the large number of unknowns this topic should be addressed in separate work.

10. Confusing terminology regarding Hill constant. Is this the output of the Hill coefficient when all theoretical values are included in the equation?

(RESPONSE) We thank Reviewer 1 for bringing this issue to our attention. Trying to understand the source of confusion we realized that the statements defining the Hill coefficients in our manuscript got deleted by mistake between different versions. We have corrected this mistake by reintroducing the missing statements (see lines 206 and 314). We apologize for this omission.

Hill coefficient and Hill constant are different factors. The Hill coefficient is the multiplicative factor in some terms of the polynomial equations. We defined the Hill coefficients in our general equations as *a* for activators and *r* for repressors. For the signaling network, the Hill coefficient for FGF was set at a value of 4 and for all other signaling factors at a value of 2, as empirically determined computationally in a somitogenesis signaling network [4]. For the transcription network, all Hill coefficients were set to a value

of 2, as the transcription factors in the pathway work as dimers [10-14]. This is a standard practice in the field (for example, see [4]).

In contrast, the Hill constant is the concentration of substrate at which rate of component interactions is half of the maximum possible value (half Vmax). In a first-order kinetic reaction (e.g., Michaelis-Menten), this value would be equivalent to the Km (Km=Vmax/2). In transcriptional factor kinetic, Hill constant, therefore, determine the affinity of the factor for the regulated downstream target. For example, a "strong" (or high affinity) transcriptional activator is required at a lower concentration (hence lower Hill constant) to achieve maximum positive regulation than a "weak" activator. In the work reported here, to analyze the operation of the signal and transcription network works, we systematically changed the values of the Hill constant to define the parametric spaces that produce different system behaviors (see Tables 1 and 2).

Specific issues raised by Reviewers 2

1. Question addressed above in the section "general issues raised by both reviewers."

2. The biggest drawback of the paper is its external validity that should be tested in an in vivo model.

(RESPONSE) We agree with Reviewer 2 that the true value of mathematical models is to test them in an *in vivo* model. We also agree with Reviewer 1 and 2 that the manuscript is quite complex and may overwhelm some of readers. In the manuscript we have identified areas where the model and the *in vivo* data do not agree, and provide possible explanations for these discrepancies (e.g., Future Perspective section, see line 679). While we have tried and failed to resolve some of these discrepancies using experimental approaches (e.g., analysis of NOTCH and CDX4 interactions; data available upon request), we feel that additional *in vivo* experiments would be better served in a follow up paper.

Supporting references

1. Waddington CH. Canalization of development and the inheritance of acquired characters. Nature. 1942;150:563-5.

2. Marom K, Shapira E, Fainsod A. The chicken caudal genes establish an anterior-posterior gradient by partially overlapping temporal and spatial patterns of expression. Mech Dev. 1997;64(1-2):41-52.

3. Schoenwolf GC. Observations on closure of the neuropores in the chick embryo. Am J Anat. 1979;155:445-465.

4. Tiedemann HB, Schneltzer E, Zeiser S, Hoesel B, Beckers J, Prezmeck GK, et al. From dynamic expression patterns to boundary formation in the presomitic mesoderm. PLoS Comp Biol. 2012;8: e04379.

5. Kiparissides A, Koutinas M, Moss T, Newman J, Pistikopoulos EM, Mantalaris A. Modelling the Delta1/Notch1 pathway: in search of the mediator(s) of neural stem cell differentiation. PLoS One. 2011;6: e14668.

6. Jurberg AD, Aires R, Varela-Lasheras I, Novoa A, Mallo M. Switching axial progenitors from producing trunk to tail tissues in vertebrate embryos. Dev. Cell. 2013;25: 451-462.

7. Aires R, de Lemos L, Novoa A, Jurberg AD, Mascrez B, Duboule D, et al. Tail Bud Progenitor Activity Relies on a Network Comprising Gdf11, Lin28, and Hox13 Genes. Dev. Cell. 2019;48:383-395.

8. Akai J, Halley PA, Storey KG. FGF-dependent Notch signaling maintains the spinal cord stem zone. Genes Dev. 2005;19: 2877-2887.

9. Denans N, Iimura T, Pourquie O. Hox genes control vertebrate body elongation by collinear Wnt repression. eLife. 2015;4: 04379.

10. Muller CW, Herrmann BG. Crystallographic structure of the T domain-DNA complex of the Brachyury transcription factor. Nature. 1997;389: 884-888.

11. Hou L, Srivastava Y, Jauch R. Molecular basis for the genome engagement by Sox proteins. Semin Cell Dev Biol. 2017;63: 2-12.

12. Gregory PA, Gardner-Stephen DA, Rogers A, Michael MZ, Mackenzie PI. The caudal-related homeodomain protein Cdx2 and hepatocyte nuclear factor 1alpha cooperatively regulate the UDP-glucuronosyltransferase 2B7 gene promoter. Pharmacogenet Genomics. 2006;16: 527-536.

13. Singh S, Stellrecht CM, Tang HK, Saunders GF. Modulation of PAX6 homeodomain function by the paired domain. J Biol Chem. 2000;275:17306-17313.

14. Gradwohl G, Fode C, Guillemot F. Restricted expression of a novel murine atonal-related bHLH protein in undifferentiated neural precursors. Dev. Biol. 1996; 180:227-241.
